# Evaluation of atezolizumab plus bevacizumab combination therapy for hepatocellular carcinoma using contrast-enhanced ultrasonography

**DOI:** 10.1007/s10396-022-01260-0

**Published:** 2022-09-28

**Authors:** Shinsuke Uchikawa, Tomokazu Kawaoka, Hatsue Fujino, Atsushi Ono, Takashi Nakahara, Eisuke Murakami, Masami Yamauchi, Daiki Miki, Michio Imamura, Hiroshi Aikata

**Affiliations:** Department of Medicine and Molecular Science, Division of Frontier Medical Science Programs for Biomedical Research Graduate School of Biomedical Sciences, Hiroshima University, 1-2-3 Kasumi, Minami-ku, Hiroshima, 734-8551 Japan

**Keywords:** Atezolizumab plus bevacizumab combination therapy, Contrast-enhanced ultrasonography

## Abstract

**Purpose:**

Previous reports suggest that contrast-enhanced ultrasonography (CEUS) is useful for predicting the efficacy of sorafenib and lenvatinib treatment. However, there are no reports on the utility of CEUS for predicting the efficacy of atezolizumab plus bevacizumab combination therapy (Atezo + Bev). This study aimed to identify CEUS parameters for predicting the efficacy of Atezo + Bev.

**Methods:**

A total of 30 patients with hepatocellular carcinoma (HCC) treated with Atezo + Bev who underwent CEUS before and 5 weeks after treatment initiation were included.

**Results:**

Post area under the curve (post AUC) was identified as a predictive factor for early progressive disease (PD). The optimal cut-off value of post AUC for predicting progression-free survival (PFS) was 61.3.

**Conclusion:**

The results of this study suggest that CEUS at 5 weeks after initiation of Atezo + Bev may predict PFS in HCC patients. Changes to the treatment plan may need to be considered in patients with post AUC > 61.3.

## Introduction

Hepatocellular carcinoma (HCC) is one of the leading causes of cancer-related mortality worldwide [1]. Hypervascularity is a known feature of HCC. Systemic chemotherapy with sorafenib and lenvatinib decreases tumor vascularity and is recommended for patients with unresectable HCC [2–4]. Previous reports demonstrated the utility of contrast-enhanced ultrasonography (CEUS) for predicting the efficacy of sorafenib and lenvatinib treatment [5–8]. The combination of atezolizumab plus bevacizumab (Atezo + Bev) was shown to result in longer overall survival (OS) and progression-free survival (PFS) than sorafenib in the IMbrave 150 trial [9], and it has become a standard for systemic first-line treatment of unresectable HCC. The present study aimed to identify CEUS parameters for predicting the efficacy of Atezo + Bev in HCC patients.

## Materials and methods

### Patients

A total of 30 HCC patients who underwent CEUS for measurement of both the tumor and background liver areas were included in the study. All patients were treated with Atezo + Bev, while some patients had received other therapies prior to Atezo + Bev treatment.

### Exclusion criteria

Patients were excluded if they (i) could not undergo CEUS before or after treatment or (ii) were lost to follow-up.

### Atezolizumab plus bevacizumab combination therapy

Patients were treated with Atezo + Bev combination therapy comprised of 1200 mg of Atezo plus 15 mg/kg of Bev. Both therapeutic agents were obtained from Chugai Pharmaceutical Co., Ltd. (Tokyo, Japan). Patients continued therapy until death or until they met one of the following criteria: (i) adverse event that required termination of treatment, (ii) deterioration of Eastern Cooperative Oncology Group (ECOG) performance status (PS), (iii) worsening liver function, or (iv) withdrawal of consent.

### CEUS imaging

CEUS was performed using an Aplio i800 imaging system (Canon Medical System, Japan) and a convex probe (3.5 MHz). Sonazoid (Daiichi Sankyo, Tokyo, Japan) was used as the perflubutane-based microbubble contrast agent. One vial of Sonazoid was dissolved in 2 ml of distilled water, and a 0.01-ml/kg solution was injected as an intravenous bolus followed by 10 ml of normal saline using a 22-gauge venous catheter that had been inserted into the cubital vein. CEUS examination was performed at baseline before initiation of Atezo + Bev combination therapy and 5 weeks later. The gain, frequency and focus position were adjusted to be the same before and after Atezo + Bev initiation.

**Fig. 1:**
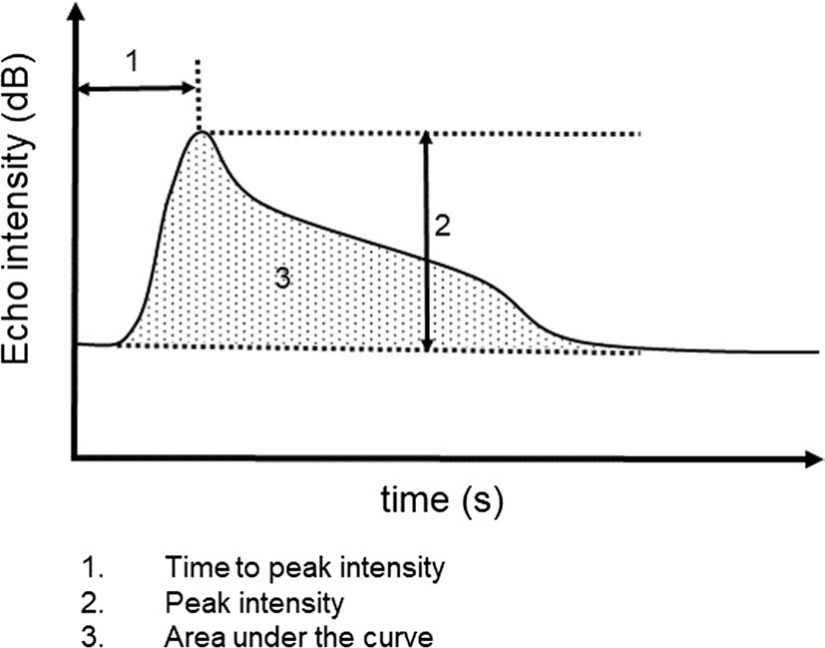
Time-intensity curve

Time-intensity curve (TIC) analysis was performed using the built-in software of the Aplio i800 imaging system. For measurement of tumor perfusion, the size of the region of interest (ROI) was adjusted to match the tumor diameter. For measurement of the liver background, a 10-mm ROI was set on the liver parenchyma. The following three TIC parameters were used for the analysis (Fig. 1): time to peak intensity, peak intensity, and total area under the TIC. The parameter before and at 5 weeks after therapy initiation was compared between progressive disease (PD) and non-PD of best response.

### Evaluation of treatment response

The radiological response was evaluated by means of computed tomography (CT) after Atezo + Bev combination therapy initiation using the mRECIST criteria. First and second evaluations were performed at 6 and 12 weeks, respectively, after initiation; subsequent evaluations were performed every 9 weeks.

### Statistical analysis

Univariate analysis was performed using the Mann–Whitney *U* test. A receiver operating characteristic (ROC) curve was calculated to evaluate the diagnostic performance of the methods to detect each grade of steatosis and stage of fibrosis. A *p*-value < 0.05 was considered statistically significant. All statistical analyses were performed using EZR (Saitama Medical Center, Jichi Medical University, Saitama, Japan), a graphical user interface for R (The R Foundation for Statistical Computing, Vienna, Austria). This modified version of R commander was designed to add statistical functions frequently used in biostatistics.

## Results

### Patient characteristics

Patient characteristics are summarized in Table 1. The median age was 69 (range, 47–83) years, and 25 (83.3%) patients were male. Of the 30 patients, four (13.3%) were HBV antigen-positive, and 11 (36.7%) were HCV antibody-positive. Eleven patients (36.7%) had macroscopic vascular invasion, and 12 patients (40%) had extrahepatic metastasis.

**Table 1:** Patient characteristics

Variable	All patients (*n* = 30)	PD at 1st evaluation (*n* = 10)	Non-PD at 1st evaluation (*n* = 20)	*p*-value
Age (range), y*	69 (66–73)	60 (55–68)	71 (68–74)	0.008†
Gender, M/F, *n*	25/5	8/2	17/3	1‡
Etiology, HBV/HCV/NBNC, n	4/11/15	3/5/2	1/6/13	0.031‡
Serum albumin (range), g/dL*	4.0 (3.4–4.2)	4.1 (3.4–4.2)	3.9 (3.4–4.2)	0.982†
Serum total bilirubin (range), mg/dL*	0.8 (0.6–1.2)	0.9 (0.6–1.0)	0.8 (0.6–1.2)	0.757†
Prothrombin activity (range), %*	96 (80–110)	95 (81–104)	97 (81–111)	0.775†
ALBI grade, 1/2a/2b/3, n	16/4/8/2	5/1/4/0	11/3/4/2	0.63‡
Child–Pugh score, 5/6/7/8/9, *n*	16/7/4/1/2	5/4/1/0/0	11/3/3/1/2	0.629‡
Extrahepatic metastasis, absent/present, *n*	18/12	4/6	14/6	0.139‡
No. of intrahepatic tumors, ≤3/ > 4, *n*	15/15	7/3	8/12	0.245‡
Tumor size relative to the liver, < 50%/≥50%, *n*	27/31	9/1	18/2	1‡
Macroscopic vascular invasion, absent/present, *n*	19/11	5/5	13/6	0.621‡
Serum α-fetoprotein (range), ng/mL*	50.7 (4.0–1136.8)	215.9 (97.2–2566.3)	26.5 (3.1–150.9)	0.022†
Serum *des*-γ-carboxy prothrombin (range), mAU/mL*	941.5 (187–7765)	351.0 (194–8348)	2589 (184–6533)	0.605†

^*^median (interquartile), †Mann–Whitney *U* test, ‡Fisher or chi-squared test

*F* female, *HBV* hepatitis B virus, *HCV* hepatitis C virus, *M* male, *PD* progressive disease

### TIC parameters

TIC parameters for the tumor (Table 2a) were as follows: median time to peak intensity pre-treatment/post-treatment: 8.7 s/8.3 s (*p* = 0.076), median peak intensity pre-treatment/post-treatment: 4.8 dB/3.0 dB (*p* = 0.017), median area under the curve (AUC) pre-treatment/post-treatment: 255.0/99.5 (*p* = 0.009). TIC parameters for the liver background (Table 2b) were as follows: median time to peak intensity pre-treatment/post-treatment: 11.2 s/14.5 s (p = 0.577), median peak intensity pre-treatment/post-treatment: 4.8 dB/3.4 dB (*p* = 0.31), median AUC pre-treatment/post-treatment: 248.1/167.5 (*p* = 0.161).

**Table 2:** TIC parameters

Variable	Atezolizumab + Bevacizumab (*n* = 30)		
Time to assessment, median (interquartile), days	34 (22–43)		
	Pre	Post	*P*-value*
(a) Tumor			
Time to peak intensity, median (interquartile)	8.7 (5.8–11.2)	8.3 (6.6–17.3)	0.076
Peak intensity, median (interquartile)	4.8 (1.2–14.5)	3.0 (1.1–6.2)	0.017
AUC, median (interquartile)	255.0 (55.1–815.5)	99.5 (30.0–333.5)	0.009
(b) Background liver			
Time to peak intensity, median (interquartile)	11.2 (7.4–20.6)	14.5 (7.6–19.0)	0.577
Peak intensity, median (interquartile)	4.8 (1.7–10.8)	3.4 (1.5–7.7)	0.31
AUC, median (interquartile)	248.1 (103.4–490.4)	167.5 (62.8–458.6)	0.161

^*^paired *t*-test,

*AUC* area under the curve

In the non-PD at first evaluation group, TIC parameters for the tumor (Table 3a) were as follows: median time to peak intensity pre-treatment/post-treatment: 9.3 s/8.4 s (*p* = 0.069), median peak intensity pre-treatment/post-treatment: 3.5 dB/2.2 dB (*p* = 0.041), median AUC pre-treatment/post-treatment: 193.3/51.1 (*p* = 0.03). There was a significant difference between pre- and post-treatment measurements for peak intensity and AUC. TIC parameters for the liver background (Table 3b) were as follows: median time to peak intensity pre-treatment/post-treatment: 12.8 s/14.6 s (*p* = 0.473), median peak intensity pre-treatment/post-treatment: 4.8 dB/4.5 dB (*p* = 0.715), median AUC pre-treatment/post-treatment: 214.6/143.1 (*p* = 0.196). There were no differences between pre- and post-treatment measurements for all TIC parameters.

**Table 3:** Comparison of changes in TIC parameters between PD and non-PD

Variable	Non-PD (*n* = 20)			PD (*n* = 10)		
	Pre	Post	*P*-value*	Pre	Post	*P*-value*
(a) Tumor						
Time to peak intensity, median (interquartile)	9.3 (6.0–11.5)	8.4 (6.5–20.2)	0.069	8.4 (5.6–10.1)	8.0 (6.7–10.0)	0.824
Peak intensity, median (interquartile)	3.5 (0.8–13.0)	2.2 (0.9–5.3)	0.041	7.5 (4.4–14.8)	5.8 (2.6–8.9)	0.239
AUC, median (interquartile)	193.3 (47.4–469.7)	51.1 (19.1–159.2)	0.03	587.6 (277.8–960.8)	316.3 (245.7–560.8)	0.085
(b) Background liver						
Time to peak intensity, median (interquartile)	12.8 (7.7–23.0)	14.6 (8.0–20.7)	0.473	9.6 (7.0–15.2)	12.9 (7.5–18.3)	0.685
Peak intensity, median (interquartile)	4.8 (1.7–11.7)	4.5 (1.3–8.9)	0.715	5.5 (2.0–7.6)	3.1 (2.4–5.1)	0.164
AUC, median (interquartile)	214.6 (118.1–522.3)	143.1 (66.1–433.8)	0.196	305.0 (28.8–408.2)	221.0 (39.2–458.6)	0.63

^*^Paired *t*-test

*AUC* area under the curve

In the PD at first evaluation group, TIC parameters for the tumor (Table 3a) were as follows: median time to peak intensity pre-treatment/post-treatment: 8.4 s/8.0 s (*p* = 0.824), median peak intensity pre-treatment/post-treatment: 7.5 dB/5.8 dB (*p* = 0.239), median AUC pre-treatment/post-treatment: 587.6/316.3 (*p* = 0.085). There were no differences between pre- and post-treatment measurements for all TIC parameters. TIC parameters for the liver background (Table 3b) were as follows: median time to peak intensity pre-treatment/post-treatment: 9.6 s/12.9 s (*p* = 0.685), median peak intensity pre-treatment/post-treatment: 5.5 dB/3.1 dB (*p* = 0.164), median AUC pre-treatment/post-treatment: 305.0/221.0 (*p* = 0.63). There were no differences between pre- and post-treatment measurements for all TIC parameters.

### Factors for PD at first evaluation and prediction of PFS

**Fig. 2:**
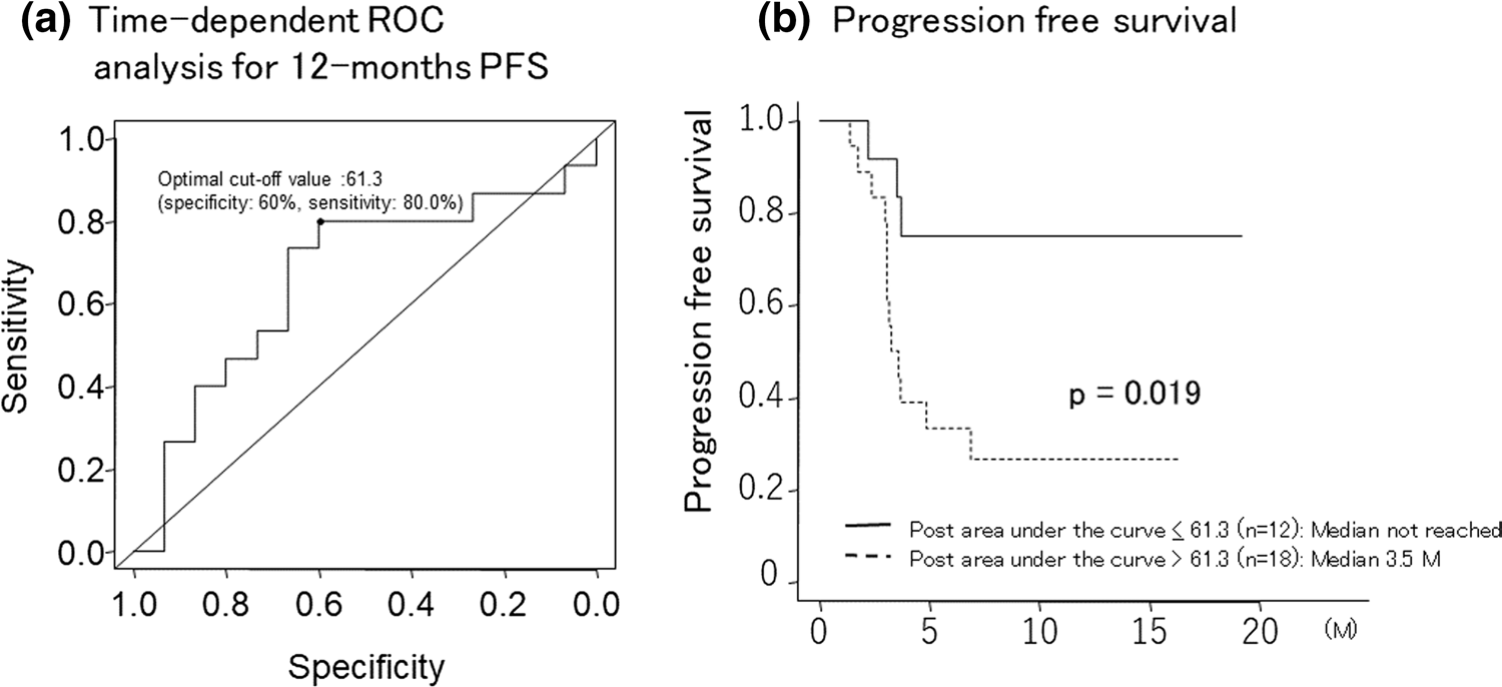
Optimal cut-off value for prediction of 12-month PFS, and comparison of PFS by post area under the curve

In the univariate analysis, predictive factors for PD at first evaluation were serum α-fetoprotein (AFP) (*p* = 0.045) and post-treatment measurement of AUC (*p* = 0.032). Multivariate analysis identified post-treatment measurement of AUC (OR 6.25, 95% CI 1.09–35.8; *p* = 0.039) as the significant predictive factor for PD at first evaluation (Table 4). Based on these results, the optimal cut-off value of post-treatment AUC for predicting 12-month PFS was determined to be 61.3 (specificity: 60%, sensitivity: 80%) according to time-dependent ROC analysis (Fig. 2a). The median PFS of the post AUC ≤ 61.3 group was significantly longer than that of the post AUC > 61.3 group (Fig. 5b).

**Table 4:** Predictive factors for PD at first evaluation

	Univariate analysis	Multivariate analysis†		
		OR	95% CI	P-value
Gender (M/F)	0.622*			
Etiology (viral/non-viral)	0.103*			
Prothrombin activity, %	0.634**			
Serum albumin, g/dL	0.75**			
Serum total bilirubin, mg/dL	0.909**			
Serum alpha fetoprotein, ng/mL	0.045**	3.03	0.50–18.5	0.230
Extrahepatic metastasis (absent/present)	0.418*			
Tumor size relative to the liver (< 50%/ ≥ 50%)	1*			
No. of hepatic tumors (≤ 3/ ≥ 4)	0.427*			
Macroscopic vascular invasion (absent/present)	0.687*			
Pre AUC (tumor)	0.226**			
Post AUC (tumor)	0.032**	6.25	1.09–35.8	0.039
Pre peak intensity (tumor)	0.497**			
Post peak intensity (tumor)	0.124**			
Ratio of post/pre AUC (tumor)	0.504**			
Ratio of post/pre peak intensity (tumor)	0.603**			

^*^Fisher or chi-squared test, **Mann–Whitney U test, †binary logistic regression analysis

AUC, area under the curve; PD, progressive disease

### Tumor response evaluation according to mRECIST criteria at 6 weeks following treatment initiation (first evaluation)

Based on the mRECIST criteria, at 6 weeks following treatment initiation (first evaluation), the proportion of patients with a complete response (CR), partial response (PR), stable disease (SD), and PD was 0% (*n* = 0), 30% (*n* = 9), 36.7% (*n* = 11), and 33.3% (*n* = 10), respectively. In the post AUC ≤ 61.3 group, CR, PR, SD, and PD were 0% (*n* = 0), 50% (*n* = 6), 41.7% (*n* = 5), and 8.3% (*n* = 1), respectively. In the post AUC > 61.3 group, CR, PR, SD, and PD were 0% (*n* = 0), 16.7% (*n* = 3), 33.3% (*n* = 6), and 50% (*n* = 9), respectively (Table 5).

**Table 5:** Efficacy of atezolizumab plus bevacizumab combination therapy at first evaluation

mRECIST (at 1st evaluation)	All patients (*n* = 30)	Post AUC < 61.3 (*n* = 12)	Post AUC > 61.3 (*n* = 18)
(a) mRECIST			
PR	9 (30.0%)	6 (50.0%)	3 (16.7%)
SD	11(36.7%)	5 (41.7%)	6 (33.3%)
PD	10 (33.3%)	1 (8.3%)	9 (50.0%)
DCR	20 (66.7%)	11 (91.7%)	9 (50.0%)
ORR	9 (30.0%)	6 (50.0%)	3 (16.7%)
(b) RECIST			
PR	3 (10.0%)	2 (16.7%)	1 (5.6%)
SD	17 (56.7%)	9 (75.0%)	8 (44.4%)
PD	10 (33.3%)	1 (8.3%)	9 (50.0%)
DCR	20 (66.7%)	11 (91.7%)	9 (50.0%)
ORR	3 (10.0%)	2 (16.7%)	1 (5.6%)

*AUC* area under the curve, *DCR* disease control rate, *ORR* overall response rate, *PD* progressive disease, *PR* partial response, *SD* stable disease

## Discussion

Atezo + Bev combination therapy is currently used as first-line systemic therapy in patients with unresectable HCC, with several molecular-targeted agents (MTAs) being used as second-line and subsequent therapy [2][2][10–12]. However, good hepatic reserve function is needed for use of MTAs, and we previously reported that HCC progression may lead to deterioration of liver function [13]. There is therefore a need to predict the efficacy of Atezo + Bev so that a change in treatment plan may be considered for patients prior to deterioration of functional reserve due to HCC progression. Bev targets vascular endothelial growth factor (VEGF), not only for immunomodulation [14] but also for angiogenesis and tumor growth, similar to VEGFR inhibition by sorafenib and lenvatinib [15][16]. The decrease in vascularity detected by CEUS is thought to reflect tumor viability [5–8]. A previous report suggests that the rate of change in TIC parameters at 7 days after lenvatinib initiation is a predictive factor for early responders [7]. In our study, the rate of change in AUC was not a predictive factor for early response to Atezo + Bev. It is possible that the AUC value measured prior to initiation of Atezo + Bev combination therapy was affected by previous therapy in some patients. In contrast, AUC at 5 weeks after initiation (post AUC) was identified as a predictive factor for early PD, regardless of previous therapy. Based on these results, the optimal cut-off value of post AUC for prediction of PFS according to time-dependent ROC analysis was determined to be 61.3. We divided the study population into two groups based on this optimal cut-off value, and found that PFS was significantly longer in the post AUC < 61.3 group compared with the post AUC > 61.3 group. This cut-off value may be a good predictor of prognosis for patients treated with Atezo + Bev combination therapy.

This study had some limitations. First, the sample size was small. Second, CEUS is an operator-dependent examination.

## Conclusion

The results of this study suggest that CEUS at 5 weeks after initiation of Atezo + Bev combination therapy may predict PFS in patients. A change in treatment plan may need to be considered for patients with post AUC > 61.3.

## Abbreviations

HCC: Hepatocellular carcinoma
TIC: Time-intensity curve
MTA: Molecular-targeted agent
AFP: α-Fetoprotein
PFS: Progression-free survival
AUROC: Area under the receiver operating characteristic curve
CEUS: Contrast-enhanced ultrasonography
ROI: Region of interest
