# Correction: Evaluation of atezolizumab plus bevacizumab combination therapy for hepatocellular carcinoma using contrast-enhanced ultrasonography

**DOI:** 10.1007/s10396-024-01430-2

**Published:** 2024-03-11

**Authors:** Shinsuke Uchikawa, Tomokazu Kawaoka, Hatsue Fujino, Atsushi Ono, Takashi Nakahara, Eisuke Murakami, Masami Yamauchi, Daiki Miki, Michio Imamura, Hiroshi Aikata

**Affiliations:** Department of Medicine and Molecular Science, Division of Frontier Medical Science Programs for Biomedical Research Graduate School of Biomedical Sciences, Hiroshima University, 1-2-3 Kasumi, Minami-ku, Hiroshima, 734-8551 Japan

**Correction: Journal of Medical Ultrasonics (2023) 50:57–62** 10.1007/s10396-022-01260-0

The article “Evaluation of atezolizumab plus bevacizumab combination therapy for hepatocellular carcinoma using contrast‑enhanced ultrasonography”, written by Shinsuke Uchikawa, Tomokazu Kawaoka, Hatsue Fujino, Atsushi Ono, Takashi Nakahara, Eisuke Murakami, Masami Yamauchi, Daiki Miki, Michio Imamura and Hiroshi Aikata, was originally published Online First without Open Access. After publication in volume 50, issue 1, page 57–62 the author decided to opt for Open Choice and to make the article an Open Access publication. Therefore, the copyright of the article has been changed to © The Author(s) 2022 and the article is forthwith distributed under the terms of the Creative Commons Attribution 4.0 International License, which permits use, sharing, adaptation, distribution and reproduction in any medium or format, as long as you give appropriate credit to the original author(s) and the source, provide a link to the Creative Commons licence, and indicate if changes were made. The images or other third party material in this article are included in the article’s Creative Commons licence, unless indicated otherwise in a credit line to the material. If material is not included in the article’s Creative Commons licence and your intended use is not permitted by statutory regulation or exceeds the permitted use, you will need to obtain permission directly from the copyright holder. To view a copy of this licence, visit http://creativecommons.org/licenses/by/4.0.

The original article has been corrected.

